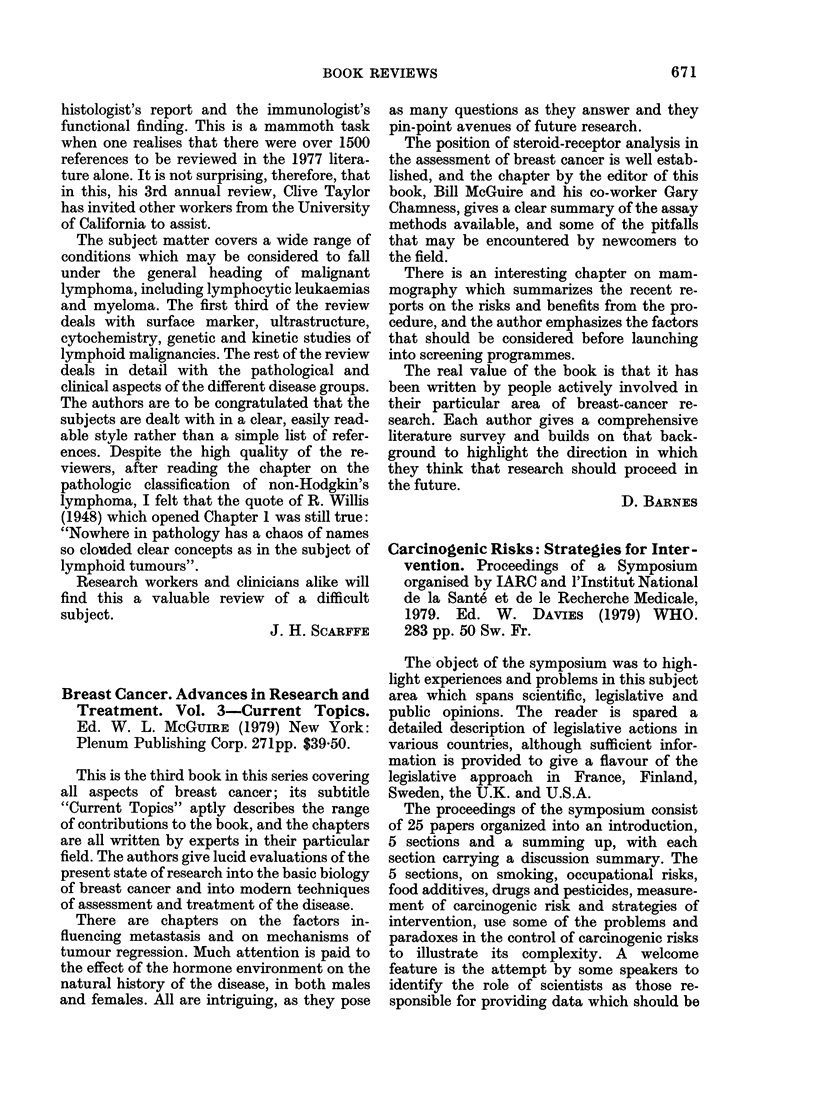# Breast Cancer. Advances in Research and Treatment. Vol. 3—Current Topics

**Published:** 1980-04

**Authors:** D. Barnes


**Breast Cancer. Advances in Research and Treatment. Vol. 3—Current Topics.** Ed. W. L. McGuire (1979) New York: Plenum Publishing Corp. 271pp. $39·50.

This is the third book in this series covering all aspects of breast cancer; its subtitle "Current Topics" aptly describes the range of contributions to the book, and the chapters are all written by experts in their particular field. The authors give lucid evaluations of the present state of research into the basic biology of breast cancer and into modern techniques of assessment and treatment of the disease.

There are chapters on the factors influencing metastasis and on mechanisms of tumour regression. Much attention is paid to the effect of the hormone environment on the natural history of the disease, in both males and females. All are intriguing, as they pose as many questions as they answer and they pin-point avenues of future research.

The position of steroid-receptor analysis in the assessment of breast cancer is well established, and the chapter by the editor of this book, Bill McGuire and his co-worker Gary Chamness, gives a clear summary of the assay methods available, and some of the pitfalls that may be encountered by newcomers to the field.

There is an interesting chapter on mammography which summarizes the recent reports on the risks and benefits from the procedure, and the author emphasizes the factors that should be considered before launching into screening programmes.

The real value of the book is that it has been written by people actively involved in their particular area of breast-cancer research. Each author gives a comprehensive literature survey and builds on that background to highlight the direction in which they think that research should proceed in the future.

D. Barnes